# Pulse protein quality and derived bioactive peptides

**DOI:** 10.3389/fpls.2025.1429225

**Published:** 2025-02-11

**Authors:** Matthew G. Nosworthy, Bianyun Yu, L. Irina Zaharia, Gerardo Medina, Nii Patterson

**Affiliations:** ^1^ Guelph Research and Development Center, Agriculture and Agri-Food Canada, Guelph, ON, Canada; ^2^ College of Pharmacy and Nutrition, University of Saskatchewan, Saskatoon, SK, Canada; ^3^ Aquatic and Crop Resource Development, National Research Council of Canada, Saskatoon, SK, Canada; ^4^ Children’s Hospital of Eastern Ontario Research Institute, Ottawa, ON, Canada

**Keywords:** pulse, protein quality, bioactive peptide, protein efficiency ratio, protein digestibility corrected amino acid score, proteomics

## Abstract

There is a growing consumer interest in sources of dietary protein that are plant-based. Pulse crops, such as lentils, beans, chickpeas, and peas, are gaining popularity due to their environmental sustainability, nutrient density, and functional attributes. The protein content and quality of pulses vary across different pulse classes and processing methods. The biological properties of the protein and the physiologically active peptides make pulse crops attractive as potentially functional or health-promoting foods. This review highlights the nutritional quality of pulse proteins as determined by the Protein Efficiency Ratio and Protein Digestibility Corrected Amino Acid Score as well as bioactive properties of specific bioactive peptides related to amelioration of hypertension and diabetes. Additionally, the use of proteomics platforms, such as mass spectrometry, in combination with bioinformatics tools, enables the identification and characterization of bioactive peptides in pulse crops. These technologies facilitate the development of pulse-derived products with enhanced nutritional values. Overall, the high nutritional quality of pulse-based proteins supports the benefits of pulse inclusion in the diet, which can also exert beneficial bioactivities resulting in improving outcomes in non-communicable diseases.

## Introduction

The dietary consumption patterns of humans are recognized as an important issue in overall health status, and also as a strategy to prevent and/or attenuate the development of several diseases (Muzquiz et al., 2012). The global management of agricultural production, as well as the adoption of alternative and sustainable food sources for the increasing world population, will require high-yielding crops with improved nutritional quality that can be productive under increasingly variable climate conditions. Recently, the nutritional content of foods has re-emerged as a key element of improving the quality of food production and diets due to increased consumer knowledge and nutrient desire. Worldwide, there is a growing shift in diet to nutritionally dense and functional foods to address health concerns (Singh and Basu, 2012). In 2018, the global market size of functional foods was about 150 billion US dollars, with this number expected to rapidly climb up to about 250 billion US dollars by 2024 (Rattanachaikunsopon and Phumkhachorn, 2018). The anticipated expansion of the market will be driven by growing effectiveness of functional foods and their bioactive components that guarantee the dietary nutritional needs.

In addition to the nutritional value of a food, or a crop, it is important to consider the bioactive components that are inherently present. Dietary proteins are known to carry a wide range of nutritional, functional, and biological properties. Nutritionally, proteins are a source of energy and amino acids essential for maintenance of growth, homeostasis, and combatting disease, among other metabolic interactions. Functionally, proteins contribute to the physicochemical and sensory properties of foods. Many dietary proteins also possess specific biological properties which make these components potential ingredients for the development of functional foods. These properties are attributed to physiologically active peptides encrypted in dietary protein sequences. These peptides are inactive, or have reduced activity, while within the native protein sequence and can be released during gastrointestinal digestion or via enzymatic exposure during food processing and germination (Korhonen and Pihlanto, 2006; Daliri et al., 2017). The size of these biologically functional peptides generally ranges between 2 and 20 amino acid residues and numerous important regulatory functions have been associated with these peptide fragments, including antihypertensive, antimicrobial, immunomodulatory, opioid, antioxidant, and mineral binding activities (Rutherfurd-Markwick, 2012; Soory, 2012; Sánchez and Vázquez, 2017). Currently, bioactive peptides (BPs) or protein hydrolysates can be commercialized as nutraceutical products or functional ingredients (López-Barrios et al., 2014).

Although many foods exhibit therapeutic potential, pulse-based foods are currently being valued worldwide due to their environmental sustainability, nutrient density, and functional attributes. Pulses such as lentils, common beans, chickpeas, and dry peas, are widely recognized for their potential to simultaneously promote human and environmental health (Foyer et al., 2016; Bessada et al., 2019). Recently there has been a growing interest in pulses and new pulse ingredients due to health promoting benefits (Aguilera et al., 2011), particularly the prevention and management of highly prevalent chronic inflammation and oxidative stress-related noncommunicable diseases (Guardado Félix and Gutierrez Uribe, 2019). In addition to the high protein content, a significant part of the potential health benefits and disease-preventing properties attributed to pulses are derived from the presence of ‘non-nutrient’ compounds that include dietary fiber, phytochemicals and antioxidants (Xu and Chang, 2007; Campos-Vega et al., 2010; Awika and Duodu, 2017; Giusti et al., 2017). Challenges therefore exist throughout the food development process, from the selection of best pulse type/cultivar (Carbas et al., 2020) to the best derived ingredients (Foschia et al., 2017). Several unique peptides generated from pulse protein isolates have been identified and associated with various functional attributes (Cosson et al., 2022; Asledottir et al., 2023). Given these points it is important to consider not only the nutritional value of a protein source such as pulses, but also the bioactivities of specific amino acids and peptides commonly found in pulses as well as their hydrolysates.

## Pulse protein content and quality

Pulse crops have long been known to be an excellent source of dietary protein compared to most vegetable-based foods (Martín-Cabrejas, 2019). As protein is a vital nutrient in foods and ingredients, it is important to consider two aspects of its nature, total protein content and its nutritional quality. Plant-based protein sources typically have lower protein content when compared to animal sources; however there exists a considerable variation across different crops. The protein content of cereals falls in the range of 8-15% depending on the species, with wheat being reported as containing 13% protein (Mathai et al., 2017), while oilseeds such as soy can be as high as 46% (Nosworthy et al., 2023). Pulse protein content falls between cereals and soy, with a range of 20-30% depending on pulse class and processing method (Hall et al., 2017; Nosworthy et al., 2018a, 2020). Much like most plant-based foods, processing pulses is necessary due to the presence of antinutritional factors such as protease inhibitors and tannins which impact the digestibility/bioavailability of nutrients present in these foods. Processing also enhances the palatability of these foods leading to a more pleasant experience during consumption.

While pulses have a relatively high protein content compared to other plant sources, the nutritional quality must also be considered. There are multiple methods assessing protein quality, with many of them involved in regulating content claims in different global jurisdictions. These methods can be divided into two groups, those that require only chemical assessment of the food/ingredient and those that necessitate animal experimentation. For foods in Europe the guidelines stipulate that a “source” of protein must provide 12% of its energy from protein and 20% for a “high source” (available online: https://eur-lex.europa.eu/legal-content/en/ALL/?uri=CELEX%3A32006R1924, European Commission, 2006). Similarly, Oceania bases their guidelines on the protein content per serving with 5 g allowing a content claim and 10 g providing a “good source” of protein available online: (https://www.legislation.gov.au/F2015L00394/2017-09-07/text, Food Standards Australia New Zealand 2015). Conversely, the Protein Efficiency Ratio (PER) and Protein Digestibility Corrected Amino Acid Score (PDCAAS), required in Canada and the United States respectively, necessitate animal experimentation.

**Figure 1:**
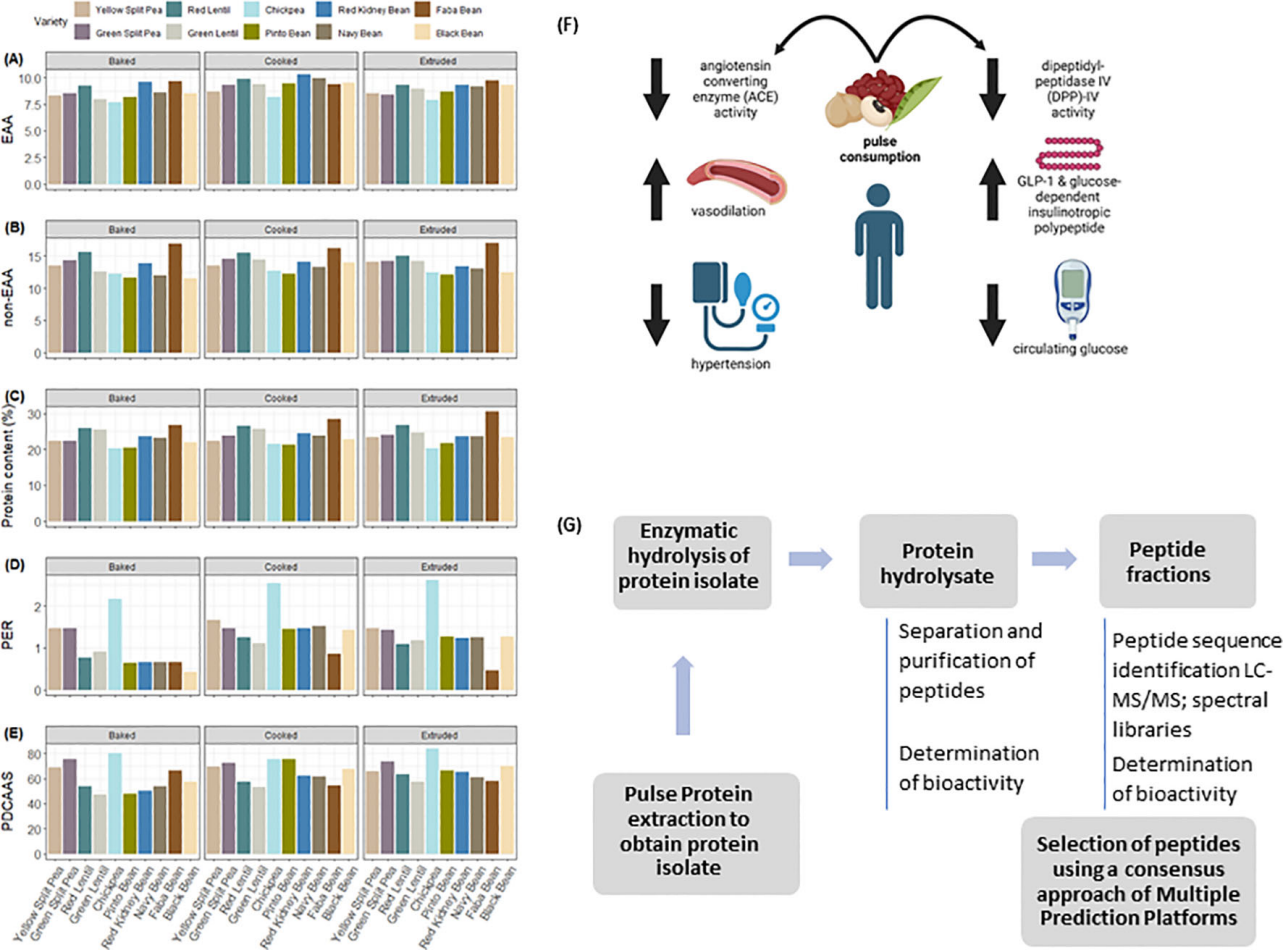
**(A)** Total essential and **(B)** non-essential amino acids in thermally processed pulses. EAA: Essential amino acids; NEAA: Non-essential amino acids. Data on processed peas were adapted from Nosworthy et al. (2017), lentils from Nosworthy et al. (2018b), chickpea from Nosworthy et al. (2020), and beans from Nosworthy et al. (2018a). **(C)** Protein content, **(D)** Protein Efficiency Ratio (PER), and **(E)** Protein Digestibility Corrected Amino Acid Score (PDCAAS) of thermally treated pulses. **(F)** Impact of pulse consumption angiotensin converting enzyme and Dipeptidyl-peptidase IV activity and downstream implications in human health. **(G)** Workflow for the identification and evaluation bioactive peptides derived from pulse protein.

The methods for determining PER and PDCAAS are well described elsewhere (Marinangeli and House, 2017). Briefly, PER is a growth measurement where the efficiency by which a rat can convert dietary protein into growth indicating the quality of dietary protein (Health Canda, 1981; available online: http://www.hc-sc.gc.ca/fn-an/alt_formats/hpfb-dgpsa/pdf/res-rech/fo-1-eng.pdf; accessed on 20 January 2024). This PER is subsequently compared to a casein control group to generate an adjusted PER score, which is then used to generate a Protein Rating where a value of ≥20 is a “good source” of protein and ≥40 is an “excellent source”. PDCAAS relies on the comparison between the amino acid composition of a food and human requirements which is then corrected by a measurement of protein digestibility determined in a rat (FAO/WHO, 1991). The PDCAAS score is then used to correct the protein content in a food serving where if the resulting corrected protein score is ≥ 5 g per serving the food is a “good source”, ≥ 10 g is an “excellent source”. Notably a recent change in Health Canada policy has allowed for the use of a PDCAAS score and a conversion factor of 2.5 to generate a PER value (Health Canada, 2023; available online: https://www.canada.ca/en/health-canada/services/food-nutrition/legislation-guidelines/policies/measuring-protein-quality-foods.html; accessed on 24 January 2024). There is a more recent method for determination of protein quality, the Digestible Indispensable Amino Acid Score (DIAAS), which is a modification of PDCAAS with updated amino acid reference patterns and consideration of individual amino acids as the nutrient rather than protein, it has yet to be adopted for regulatory purposes by any jurisdiction (FAO/WHO, 2013). Relative essential and non-essential amino acids presented in **Figures 1A, B** and the examples of pulse protein content and quality under different processing conditions are presented in **Figures 1C-E**. The essential amino acid content of the various pulse classes and processing methods remains consistent, with variation being more present among the non-essential amino acids. While still under review (Health Canada, 2023), use of alternative conversion factors between PER and PDCAAS may be advisable depending on the protein source and processing method.

## Bioactivity of amino acids

In addition to the direct nutritional value of pulse protein, it has been well established that dietary amino acids can influence various aspects of metabolism including muscle growth, immune response, and prevention of non-communicable diseases such as hypertension and cardiovascular disease (Pedroche et al., 2002; Hong et al., 2008; Garcia-Mora et al., 2015). Many studies have investigated the biological activities of individual amino acids/peptides and the topic has been discussed in multiple reviews (Wu, 2009, 2010; Zaky et al., 2022). Individual amino acids have been demonstrated to have a wide range of functions including regulation of cell division and gene expression, nucleotide synthesis, regulation of protein turnover, and intracellular signaling among other activities (Wu, 2009). These activities are related to the individual amino acid, but during metabolic and enzymatic activities these amino acids can be converted into other compounds, such as creatine (tripeptide of arginine, glycine and methionine), glutathione (cystine, glutamine, and glycine), and taurine (formed from cystine), which are all antioxidants, as well as exhibiting other biological effects. This highlights that it is not simply the essential amino acids that need to be considered when investigating the bioactivity of different proteins and their constituent parts. Of particular interest in pulses are the amino acids arginine and leucine due to their high amounts in pulses and multifaceted bioactive effects.

Pulses are known to have a high arginine content, in particular chickpeas and fava beans (Boye et al., 2010; Bessada et al., 2019). Beyond protein synthesis, arginine is involved in many physiological processes including the synthesis of creatine, polyamines, and nitric oxide (NO) which is a known vasodilator (Tuteja et al., 2004; Maeda et al., 2006; Wu et al., 2021). Multiple studies have demonstrated that consumption of pulses lowers the incidence of cardiovascular disease and hypertension which may be tied to the higher arginine content and its function in vasodilation and cellular signaling, or the generation of bioactive peptides targeting angiotensin converting enzyme (Roy et al., 2010; Padhi and Ramdath, 2017). Leucine is a member of the branch-chain amino acids which also includes isoleucine and valine, and has been implicated in stimulating protein synthesis and muscle anabolism, as well as multiple immunomodulatory effects and attenuation of glucose response (Bonvini et al., 2018; Kamei et al., 2020). This is of particular importance in elderly individuals where a higher leucine content of a protein source has been demonstrated to enhance myofibrillar protein synthesis (Devries et al., 2018). Importantly, while content claims can be made for protein content as described previously, no regulatory body accepts health claims based on any specific amino acids (EFSA Panel on Dietetic Products, 2010; Krasniqi et al., 2016; Roberts, 2016). While this highlights the bioactivities of two amino acids with high concentration in pulses, it is the biological activity of peptides derived from pulse protein that has been the focus of significant amounts of research in recent years.

## Pulse protein and derived bioactive peptides

Recent studies suggest that protein hydrolysates and bioactive peptides derived from pulses are among the most promising bioactive proteins (Guardado Félix and Gutierrez Uribe, 2019) thus positioning these legumes as excellent sources for the development of new protein-derived products. Bioactive peptides are small amino acid sequences derived from food proteins through *in vivo* or *in vitro* enzymatic proteolysis of the inactive precursor proteins (Udenigwe and Aluko, 2012) that can hold physiological properties (López-Barrios et al., 2014). Within the native protein, the amino acid sequence conforming the bioactive peptide is either inactive or exhibits diminished activity. However, once the peptide is released it can display diverse biological activities (Udenigwe and Aluko, 2012). Accumulating evidence from *in vitro* and *in vivo* studies indicate that pulse-derived bioactive peptides can be active toward several chronic diseases like autoimmune disorders, cardiovascular disease and diabetes, and have been used as opiates, immunomodulators, antimicrobials, antioxidants, antihypertensives and antithrombotic agents (Duranti, 2006). Many bioactive peptides have common structural properties, including a relatively short residue length, hydrophobic amino acid residues, and the presence of aromatic side chains, mainly with arginine, lysine or proline in the C-terminal position (Marambe and Wanasundara, 2012; López-Barrios et al., 2014). The biological and pharmacological properties of pulse protein-derived bioactive peptides have been recently reviewed (Tak et al., 2021; Fan et al., 2022). Of particular interest are the peptides demonstrating either ameliorative or protective effects on non-communicable diseases such as diabetes and cardiovascular disease (**Figure 1F**). Selected peptide sequences demonstrated to have inhibitory activities on angiotensin converting enzyme and dipeptidyl peptidase IV are presented in **Table 1**.

**Table 1:** Bioactive peptides from pule protein sources and their function.

Bioactive function	Protein source	Peptide sequence	Reference
ACE Inhibition	Common Beans Peas Lentils Chickpeas Faba bean	PVDDPQIH, KKSSG, LSFNT, KMARPV, KYMKS, SGSYS, CPGNK, INQGSLLPH, FVVAEQAGNEEQFE GGSGNY, DLKLP, GSSDNR, MRDLK, HNTPSR KLRT, TLHGMV, VNRLM MD, MFDL, MDLA, MDFLI, MDL EEEDEDEPR, KEEEDEDEPR, VIPTEPPH, VIPTEPPHA, VVIPTEPPH, VVIPTEPPHA, NYDEGSEPR, PVNRPGEPQ, LDNINALEPDH	Rui et al. (2013); Jakubczyk et al. (2017); Mojica et al. (2017); Jakubczyk and Baraniak (2014); Jakubczyk and Baraniak (2013); Pedroche et al. (2002); Yust et al. (2012); Martineau-Côté et al. (2022)
DPP-IV Inhibition	Common Beans Peas Chickpeas Faba bean	KKSSG, LSFNT, KMARPV, KYMKS, SGSYS, CPGNK EPF, SPGDVF, IPYWTY AIPPGIPYW, PGIPYW VIPTEPPH, VIPTEPPHA, VVIPTEPPH, VVIPTEPPHA, NYDEGSEPR, PVNRPGEPQ, LDNINALEPDH	Mojica et al. (2017); Zhang et al. (2022, 2023); Zan et al. (2023); Martineau-Côté et al. (2022)

## Anti-hypertensive peptides (ACE inhibitors)

Hypertension, a known risk factor for cardiovascular disease, can be treated with the administration of drugs inhibiting an enzyme known as Angiotensin I-Converting Enzyme (ACE). This enzyme is responsible for the hydrolysis of angiotensin I to angiotensin II, which is a potent vasoconstrictor inducing elevated blood pressure. As hypertension can also be managed by alterations in diet, as well as exercise regimes, there is an interest in identifying compounds present in foods that can modify metabolic enzymes such as ACE. In the case of pulses there are multiple peptides that have been identified to inhibit ACE activity, thereby potentially leading to a reduction in blood pressure. A study investigating chickpea and pea hydrolysates demonstrated that desi and kabuli chickpeas, as well as yellow peas, are capable of inhibiting ACE activity (Barbana and Boye, 2010). Interestingly the enzymes used for hydrolysis impacted the IC_50_ for the protein hydrolysates with a range of 229-316 µg/mL for kabuli chickpea, 140-228 µg/mL for desi chickpea, and 128-412 µg/mL for yellow pea. For both types of chickpeas the use of a gastrointestinal digestion resulted in the lowest IC_50_ while papain digestion generated the lowest value for yellow peas, highlighting the importance of identifying the appropriate conditions depending on initial ingredient. A recent investigation into faba beans synthesized peptides based on those identified after *in vitro* gastrointestinal digestion and determined the differences in ACE inhibition based on peptide sequence (Martineau-Côté et al., 2024). While multiple peptides did not have any impact on ACE activity, four sequences were identified to have IC_50_ values between 43-118 µg/mL. Common among these peptides was the sequence VIPTEPPH, with the peptide having the lowest IC_50_ value of 43 µg/mL being VVIPTEPPH. In combination, this supports the idea that proper selection of initial ingredient combined with processing methods to generate a peptide with known activity can impact the development of hypertension.

## Diabetic prevention peptides (DPP-IV)

Diabetes is a well-known non-communicable metabolic disorder characterized by inadequate production of insulin or development of insulin resistance thereby leading to reduced control over glucose homeostasis. While there are multiple methods of treatment available for individuals with diabetes, ranging from a modification of diet to oral and injectable drugs, one such treatment is inhibition of the enzyme dipeptidyl peptidase-IV, or DPP-IV (Silveira et al., 2013). This enzyme is a serine protease responsible for the degradation of glucagon-like peptide-1, as well as glucose-dependent insulinotropic polypeptide. As these two incretins are responsible for stimulating insulin release and decreased glucagon release, DDP-IV activity results in an increased blood glucose concentration, a concern for diabetics. The ‘-liptin’ group of drugs such as sitagliptin are an approved drug for diabetic treatment that target this enzyme. Similar to that of ACE, there has been great interest in identifying peptides present in foods such as pulses that can also inhibit the activity of DPP-IV. One study investigated the generation of bioactive peptides targeting DPP-IV in post-simulated gastrointestinal digestion from pulses via milling, thermal treatments, fermentation, and germination (Di Stefano et al., 2019). The combination of heat treatment and fermentation increased DPP-IV inhibition in chickpeas, while fermenting ground green lentils and yellow peas induced increase of DPP-IV inhibition compared to other treatments investigated. Another study investigated the use of different digestive enzymes on the generation of DPP-IV inhibitory peptides from chickpea protein (Zan et al., 2023). In this case, application of Neutrase or Papain resulted in the greatest inhibition of DPP-IV at approximately 47% and 35% inhibition respectively. A time-course experiment determined that a digestion time of 60 minutes with Neutrase generated the highest inhibitory activity (52.50 ± 0.88%). During this experiment the IC_50_ values for the identified inhibitory peptides ranged from 12.43 µM, IAIPPGIPYW, to 110.50 µM, LAFP, with the positive control peptide exhibiting the greatest inhibition, diprotin, reaching 6.81 µM as a point of comparison. As there are a wide range of protein fragments and peptides generated by processing and digestion, it is crucial to employ methods for identification of these peptides and characterization of cultivars that can generate greater concentrations of bioactive peptides.

## Proteomics platforms for analysis of pulse protein-derived bioactive peptides

The scientific advances in ‘OMICS’ technologies combined with molecular biology approaches in recent years have impacted crop breeding and food science significantly (Varshney et al., 2009; Kato et al., 2011). In particular, the application of these technologies to identify and select crop varieties that offer wider functional scope, enhance the nutritional profile of seed/grain and possess bioactive potential, is of significant interest to farmers, consumers, and product developers. OMICS technologies are driving a knowledge revolution toward a comprehensive understanding of functional attributes of foods, particularly the variability of bioactive molecules composition in food production and processing to improve nutritional value and health. Ahmed et al. (2022) emphasized three key advances in omics technology enabling unprecedented food composition analysis and application: (i) high-throughput platforms for analysis of a broad range of food molecules; (ii) high-resolution biochemical libraries; and (iii) data integration and machine learning.

Generally, analysis of plant protein-derived peptides is based on high-throughput proteomics platform to comprehensively profile untargeted and targeted peptides using mass spectrometry (MS) techniques, nuclear magnetic resonance, and bioinformatics tools. MS has arguably become the core technology for the qualitative and quantitative analysis of food proteins and peptides, and providing understanding of their nature, structure, functional properties and impact on human health. Interfacing of high-resolution MS for food protein analysis with robust standard reference chemical libraries has enabled the identification of peptides based on their mass spectra with a high degree of accuracy and confidence. Some of the techniques used for bioactive peptides purification and identification have been recently reviewed by Du and Li (2022). The emergence of open-source protein databases (reviewed by Naeem et al., 2022) offers comprehensive spectral libraries that increase the efficiency of identifying bioactive molecules including molecular weight and structure.

To exploit the functional value of peptides, sequential purification and identification of various peptides are routinely used (Singh et al., 2023). This review is intended to highlight some of the MS-based strategy applications in pulse crops research defining structure/functional relation of protein and derived peptides. Commonly, ultrafiltration or in combination with chromatographic techniques allows protein hydrolysates to be separated into different fractions with specific molecular weights (MWs) for the determination of their biological activities (**Figure 1G**). In a study by Li and Aluko (2010), purified peptide fractions from pea protein isolate were analyzed by ultra-performance liquid chromatography-tandem mass spectrometry (UPLC-MS/MS) to identify and obtain amino acid sequence of the most abundant peptide in each peak. The major peptides identified showed strong inhibitory properties toward ACE and renin that may be potentially useful as ingredients to formulate multifunctional food products and nutraceuticals. In a recent study to establish a peptide profile and identify specific pea protein-derived peptides that suppressed glucose production in mouse liver cell-line AML-12 cells, Liao et al. (2022) analyzed pea protein hydrolysate using a nano-UPLC coupled with a Q Exactive HFX Orbitrap instrument. The MS spectra of most of the peptides within the range of molecular weight of 1000 to 1499 kDa were processed using the Proteome Discoverer (PD) software (Version 2.4.0.305, Thermo Fisher Scientific) and the built-in Sequest HT search engine. The sequences were searched for in the UniProt FASTA databases (uniprot-Pisum sativum_3888.fasta). Consequently, this study revealed a new function of pea protein hydrolysate in its ability to exhibit anti-diabetic activity. A study by Karkouch et al. (2017) aimed to identify peptides with antioxidant, antityrosinase and antibiofilm activities released from *Vicia faba* (dry broad bean) seed proteins hydrolysate. The purified fraction was analyzed by liquid chromatography mass spectrometry (LC–MS/MS) coupled with an LTQ-Orbitrap hybrid mass spectrometer equipped with a nano-ESI source and annotation of peptide sequence based on MS/MS spectra was performed using the Peaks software (BSI, Canada). As such, seven peptides were identified and further demonstrated their potential as a natural source of bioactive peptides for applications in the cosmetic and pharmaceutical industries. A recent review by Hou et al. (2023) provided a comprehensive review of MS-based technologies for identification of peptides purified from mung bean, their biological activities, and their potential applications. Although there are some variations in MS equipment, applications of these instruments, the integration of large spectral datasets with bioinformatics has enabled and advanced quantitative determination and identification of pulse protein-derived peptides (**Figure 1G**). The review by Du and Li (2022) also highlights the available web-based tools to evaluate the relationship between the function and structure of bioactive peptides. Models based on quantitative structure/activity relationships (QSAR) and quantitative structure/property relationships (QSPR) are employed to evaluate specific bioactive peptide activities quantitatively (by QSAR) or qualitatively (by QSPR), using unknown activity data and structural information. In particular, PeptideRanker is widely used QSPR to predict the activity of different peptides by integrating peptides sequence datasets and structural parameters. The utilization of bioinformatics tools is essential when employing various MS-based technologies as it offers opportunities for the pulse industry to deploy products with enhanced nutritional values.

## Conclusion

Pulses are a staple food in many regions of the world with a rising consumer interest in the Western world due to their nutritional density and environmental sustainability. Although the protein content and overall nutritional quality of pulses is high compared to other crops such as cereals, pulse class selection and method of processing can significantly impact the overall quality. This is particularly notable in the case of baked beans as they did not stimulate growth at a comparable rate to other thermal processing methods. In addition to their high nutritional quality, pulse protein also possesses numerous bioactive properties. Of particular interest is their inhibitory properties on ACE and DPPIV leading to a reduction in the severity of hypertension and diabetes. Identification and characterization of these bioactive peptides using modern technologies and tools such as proteomics, bioinformatics, peptide libraries and *in vitro* assays, will lead to the development of functional food ingredients and nutraceutical products with enhanced health benefits.
